# Pro re nata versus fixed aflibercept regimen for neovascular age-related macular degeneration: a systematic review and meta-analysis

**DOI:** 10.1186/s40942-022-00416-x

**Published:** 2022-09-22

**Authors:** Andi Arus Victor, Yan Martha Putri

**Affiliations:** 1 Department of Ophthalmology, Faculty of Medicine, Universitas Indonesia, Dr. Cipto Mangunkusumo National General Hospital, Jakarta, Indonesia; 2 Faculty of Medicine, Universitas Indonesia, Dr. Cipto Mangunkusumo National General Hospital, Jakarta, Indonesia

**Keywords:** Aflibercept, Pro re nata, Bimonthly, Neovascular age-related macular degeneration, Age-related macular degeneration

## Abstract

**Background:**

Aflibercept is a relatively new anti-VEGF used to treat neovascular age-related macular degeneration (AMD). The purpose of this review is to evaluate the effect of pro re nata (PRN) and fixed regimen (bimonthly) of aflibercept injection for neovascular AMD on visual outcomes at 12 months of follow-up.

**Methods:**

We conducted a systematic search in PubMed (MEDLINE), Embase, Scopus, and Web of Science, EBSCOHost, and ClinicalTrials.gov databases. Number of injections, number of hospital visit, mean change of best corrected visual acuity (BCVA), mean change of central macular thickness (CMT), and adverse effects of the included studies were evaluated. Meta-analysis were performed using Review Manager 5.4.

**Results:**

Four studies were selected for meta-analyses synthesis (3 RCT, 1 retrospective study). A total of 197 eyes in PRN group and 241 eyes in the fixed group. All four studies favored fixed regimen with standardized mean difference of 0.56 (95% CI 0.36–0.75, I^2^ = 0%, *p* < 0.00001). There was no significant difference in CMT between both group with SMD of 0.17 (95% CI − 0.14–0.48, I^2^ = 26%, *p* = 0.28).

**Conclusion:**

The present meta-analysis shows that bimonthly injection of aflibercept for neovascular AMD is superior compared to PRN injection, shown by better improvement in BCVA at 12 months follow-up. However, high risk of bias downgrade the certainty of evidence.

**Supplementary Information:**

The online version contains supplementary material available at 10.1186/s40942-022-00416-x.

## Introduction

Age-related macular degeneration (AMD) has been a debilitating eye disease that causes vision loss in the elderly population [1]. Wong et al. [2] had projected that in 2020, 196 million people which accounts for 8.7% of the world population will develop AMD and the number will continue to increase until 2040 to as much as 288 million people [2].

It has been known that inflammatory process plays a big role in the pathogenesis of CNV. Oxidative stress, light exposure, autoimmune mechanism, and even deficiency in diet had been hypothesized to generate inflammation that leads to drusen formation [3, 4]. The inflammation process ultimately causes imbalance between proangiogenic growth factors and anti-angiogenic factors, in which proangiogenic growth factors such as the vascular endothelial growth factor (VEGF) increases while the anti-angiogenic factors decreases. Thus, stimulating angiogenesis. Overexpression of VEGF-A in retinal pigment epithelium (RPE) and vitreous had been found numerously in previous studies. In comparison with other VEGF subtypes, VEGF-A is believed to be the most important VEGF in the development of CNV. Hence, anti-VEGF administration has been the mainstay of pharmacological treatment in wet AMD [4, 5].

Aflibercept is a relatively new anti-VEGF that binds VEGF-A and also placental growth factor (PIGF). With this drug having higher affinity for binding compared to its natural receptor, aflibercept is able to inhibit the stimulation angiogenesis. In comparison with other anti-VEGF such as ranibizumab and bevacizumab, aflibercept has stronger affinity towards VEGF. This pharmacokinetic of aflibercept makes it conceivably having a longer duration of action in the eye [5–8]. Thus, leading to less injections needed and less adverse events experienced by the patients.

The recommended dose of aflibercept injection for neovascular AMD according to the printed label is an injection of 2 mg aflibercept every 2 months. However, other dosing protocols are also used by ophthalmologist such as *pro re nata* (PRN) or as needed protocol and treat-and-extend protocol. Recent review on anti-VEGF dosing regimens for neovascular AMD by Li et al. [9] revealed that monthly injections of anti-VEGF exerted better visual outcome and lesser adverse events compared to PRN regimen. Nevertheless, due to high-cost burden on monthly injection of anti-VEGF, the PRN regimen is still considered reasonable.

The purpose of this review is to evaluate the effect of PRN and fixed regimen of aflibercept injection for neovascular AMD primarily on visual outcome at 12 months of follow-up. The result of this study can aid clinicians on choosing the suitable dosing regimen for patients with neovascular AMD.

## Methods

### Protocol and registration

Prior to the writing of this review and meta-analysis, we submitted a protocol, and it was registered in the International prospective register of systematic reviews (PROSPERO) on May 21, 2021 (CRD42021250407). We formulate this review in reference to the Preferred Reporting Items for Systematic Reviews and Meta-Analyses (PRISMA) checklist [10].

### Eligibility criteria for study selection

Retrospective cohort studies and clinical trials that evaluate pro re nata or as needed regimen as compared to fixed or scheduled regimen of aflibercept in patients with treatment naïve neovascular AMD are included in this study. Studies that evaluate the effect of switching anti-VEGF therapy or patients with prior pharmacotherapy and surgical treatment were excluded. Studies without available full-text and written in other than English or Indonesian language were also excluded. The main outcome of this study is the standardized mean difference (SMD) of best corrected visual acuity (BCVA) at 12 months of follow-up, recorded in Early Treatment Diabetic Retinopathy Study (ETDRS) letters or converted to ETRDRS letters. The secondary outcomes include mean change of CMT, total number of aflibercept injections, hospital visit, and adverse effects.

### Search methods for identifying studies

A systematic search was conducted in PubMed (MEDLINE), Embase, Scopus, and Web of Science, EBSCOHost, and ClinicalTrials.gov databases was performed from the inception of databases up until 1 May 2021 to identify relevant studies, using the following keywords to maximize the search including: “aflibercept”, “pro re nata”, “macular degeneration”, “as needed”, “retrospective”, and “trial”.

### Study selection

Systematic searching from the aforementioned electronic databases was conducted and the results were exported to reference manager software Mendeley. Duplicate articles will be removed. Two reviewers (YMP, AAV) screened for article titles and abstracts independently. The reminder articles were then screened for full-text eligibility by the two reviewers independently. Disagreements between reviewers were resolved through discussion.

### Risk of bias assessment

Risk of bias assessment was performed using Cochrane Collaboration’s tool for assessing the risk of bias in randomized trials for RCT studies and the modified Newcastle–Ottawa Scale (NOS) for retrospective cohort studies. For RCT studies, the risk of bias was assessed according to these domains; selection bias, performance bias, detection bias, attribution bias, reporting bias, and other bias. Then the articles will be categorized into ‘low risk’, ‘high risk, or ‘unclear risk’ of bias for each domain. For cohort studies, the risk of bias was assessed through its selection, comparability, and outcome. Maximum point that can be allocated was 9 and studies with a score of 6 and above were considered as high-quality studies. Any disagreements between reviewers will be resolved through discussion or by consulting a third reviewer.

### Data extraction and synthesis

Data extraction was performed from the included articles. Data extracted were: first author, year of publication, study location, study design, number of enrolled subjects, age, total number of injections, number of hospital visit, BCVA before treatment, BCVA after treatment, treatment details (regimen, duration, and dose), risk of bias, duration of follow-up, and adverse effects. For missing or incomplete data, the corresponding author of the study was contacted through e-mail.

### Statistical analysis

The summary measure of primary outcome will be the difference in mean change of BCVA from baseline in PRN and fixed group. Studies without known standard deviation (SD) were calculated manually. Studies using median and range were considered as normally distributed. Review Manager 5.4 was utilized to analyze the data from included studies and create meta-analysis. Effect measures for continuous data was used to calculate SMD using inverse variance statistical method between the two groups with 95% confidence interval (CI) as effect measure. Analysis method used was random effects to identify any heterogeneity between studies. Statistical heterogeneity between studies was quantified using I^2^ statistics. I^2^ > 30% was considered to be moderately heterogen, substantial heterogeneity if I^2^ > 50%, and considerable heterogeneity when I^2^ > 75%. Possible sources of heterogeneity were assessed for any studies with substantial or considerable heterogeneity. We were unable to generate sensitivity analysis due to insufficient number of included studies.

## Results

### Search selection

**Fig. 1:**
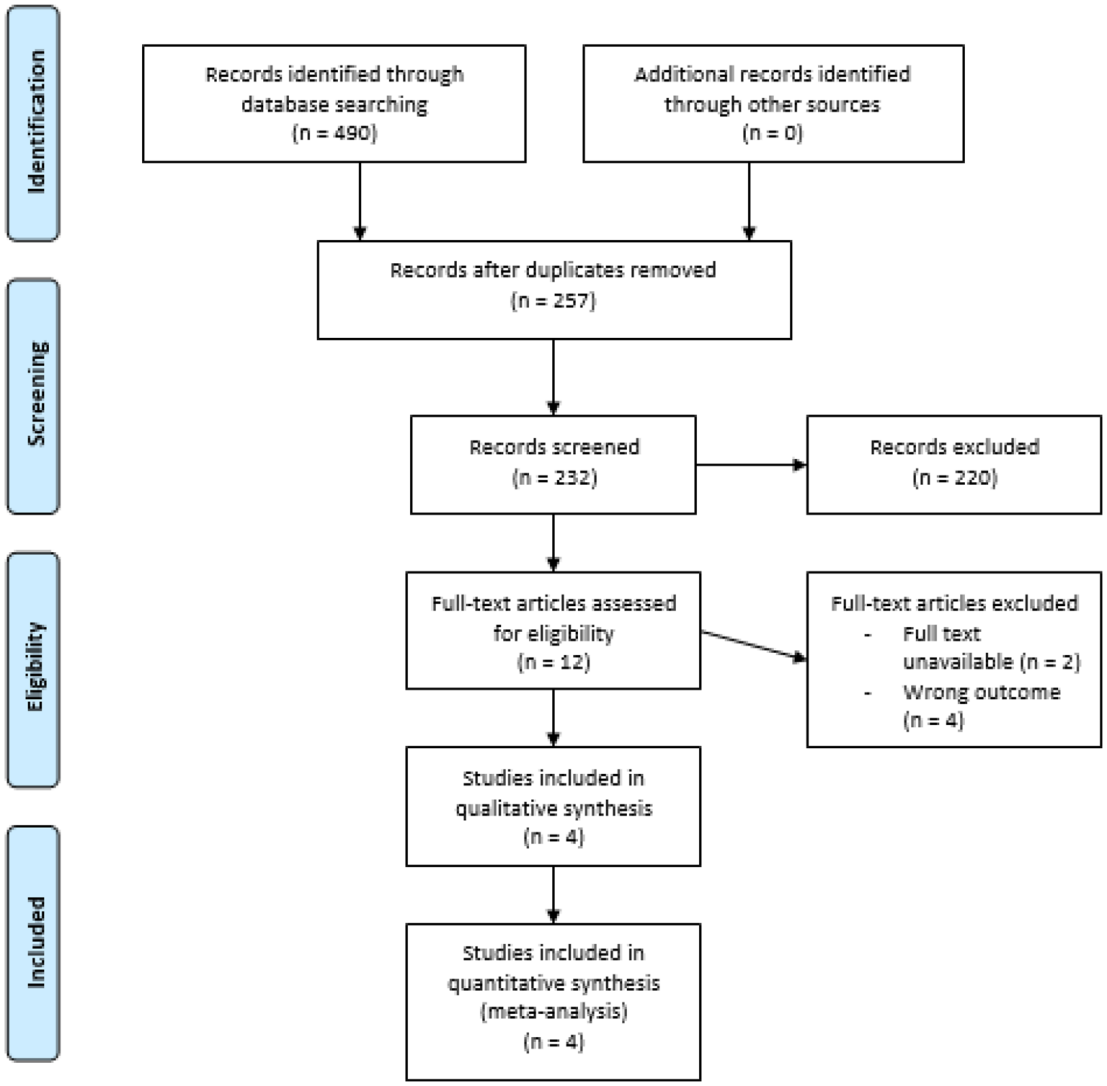
Prisma flow diagram for study selection

Prisma flow diagram for study selection is shown in Fig. 1. Initial study search resulted in 490 records from the electronic databases and one record from hand-searching. The final search for studies yielded 4 articles that were qualified to be included in this review and with sufficient outcome data to be extracted for the synthesis of meta-analyses. The most common reason for study exclusion were wrong outcome measurement, comparisons, and intervention. The four selected articles for meta-analyses synthesis were consisted of 3 controlled trials [17, 28, 29] and one retrospective cohort study [11].

### Characteristics of studies

**Fig. 2:**
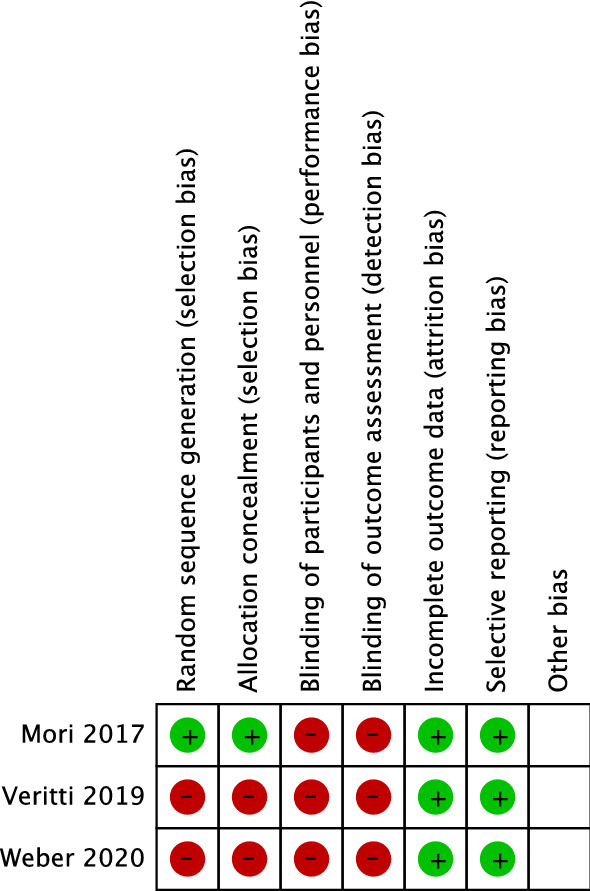
Risk of bias assessment of all included randomized trials using Cochrane Collaboration’s tool

Study characteristics are summarized in Table 1. Two studies were conducted in France [11, 12], one study was conducted in Japan [13], and one study was conducted in Italy [14]. Treatment groups were PRN or as needed aflibercept injection, whereas for the fixed group, all studies followed the dosage that is instructed on the label (one 2 mg aflibercept injection every 2 months). The total number of eyes included was 197 eyes in PRN group and 241 eyes in the fixed group. All four included study was able to be extracted for primary outcome analyses. However, we were unable to extract the necessary data for secondary outcome analyses. Adverse events that were related to treatment was only reported by Weber et al. [12] that accounted for 10% of study subjects. Risk of bias assessment for RCTs is shown in Fig. 2 and in Table 2 for the risk of bias assessment of retrospective study. We assessed high risk of bias on the selection domain in Veritti et al. [14] and Weber et al. [12] due to the absence of patient randomization and the concealment of patient allocation. As the only retrospective study in this review, Keppi et al. [11] acquired 8 scores on the Newcastle–Ottawa Scale, which means low risk of bias.

**Table 1:** Characteristics of included studies

Author (year)	Country	Study design	Age (years)*	Duration of wet AMD (months)*	Regimen	Sample (N)	BCVA at baseline (ETDRS)*	Mean change BCVA at 12-months (ETDRS)	Mean change CMT (μm)	Total injections*	Total hospital visits*	Adverse events (%)
Weber (2020)	France	RCT	79.6 ± 7.9	1.4 ± 8.7	Bimonthly	102	57.3 ± 17.9	+ 7.6	n/a	7.2 ± 0.8	9.5 ± 1.8	5
					Pro re nata	60	56 ± 24.3	+ 0.1	n/a	5.2 ± 1.8	8.8 ± 1.8	2
Veritti (2019)	Italy	RCT	77.3 ± 7.0	n/a	Bimonthly	92	56 ± 13.9	+ 6.7	− 97.362	7	4	n/a
			78.2 ± 6.2		Pro re nata	92	53.4 ± 15.2	+ 1.9	− 55.462	5.5 ± 1.6	12	
Mori (2017)	Japan	RCT	72.8 ± 2.59	n/a	Bimonthly	28	65.75 ± 4.53	+ 7.1	− 116.333	7	12	n/a
			76.5 ± 2.64		Pro re nata	30	67.43 ± 4.59	+ 3.4	− 101.333	4.8 ± 1.5	12	
Keppi (2017)	France	Retro-spective	78.4 (61–95)	n/a	Bimonthly	19	57.2 (20–72)	+ 8.4	− 113.333	7.5	4	n/a
			85.2 (62–93)		Pro re nata	15	58.4 (28–80)	− 0.4	− 105.333	7.6 (3–11)	7.8 (6–11)	

*AMD* Age-related Macular Degeneration, *BCVA* Best Corrected Visual Acuity, *ETDRS* Early Treatment Diabetic Retinopathy Study letters, *CMT* Central Macular Thickness

^*^Value in mean ± SD or median (range)

**Table 2:** Risk of bias assessment of included retrospective cohort study using Newcastle–Ottawa Scale

Author (year)	Selection	Comparability	Outcome	Total NOS Scale
Keppi (2017)	***	**	***	8

Mori et al. [13], Veritti et al. [14], and Weber et al. [12] does not report difference in mean participant age, while Keppi et al. [11], although does not report any *p*-value, have slightly older mean age in the PRN group compared to the bimonthly group (85.27 (range: 62–93) vs 78.47 (range: 61–95)). There was no sensitivity analysis in all the studies for interaction with age. Baseline BCVA was not significantly different between PRN and fixed regiment in all the studies. Weber et al. [12] did not report *p*-value of baseline BCVA between groups.

### BCVA at 12 months of follow-up

**Fig. 3:**
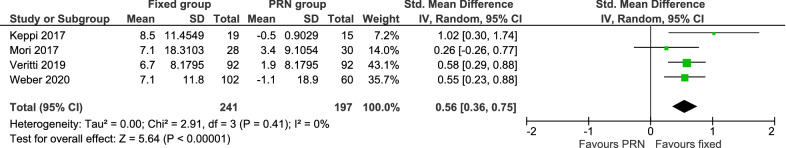
Forest plot for SMD of BCVA at 12 months of follow-up

The change of BCVA from baseline to 12 months of follow-up was calculated as SMD. All four studies favored fixed regimen of aflibercept compared to PRN regimen (Fig. 3). The pooled SMD of the four studies was 0.56 (95% CI 0.36–0.75). The forest plot resulted in no heterogeneity between the included studies (I^2^ = 0%, *p* < 0.00001). The first sensitivity analysis was conducted by excluding the only retrospective study in this review [11]. The adjusted pooled SMD was 0.52 (95% CI 0.32–0.72) (Additional file 1: Figure S1) which favored fixed aflibercept regimen. There was also no heterogeneity found (I^2^ = 0%, *p* < 0.00001). However, for the second sensitivity analysis by excluding studies with higher risk of bias [12, 14], the result demonstrated substantial heterogeneity with no statistical significance (I^2^ = 65%, *p* = 0.12, Additional file 1: Figure S2).

### Central macular thickness change from baseline

**Fig. 4:**
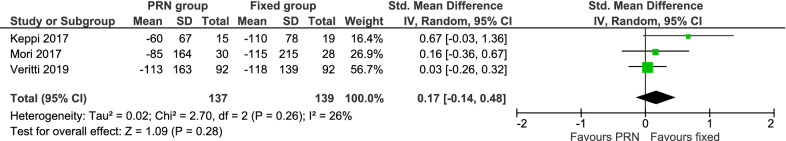
Forest plot for SMD of CMT change

Three studies [11, 13, 14] included central macular thickness (CMT) change after aflibercept treatment. The result of all the studies (Fig. 4) shows that there was no statistically significant difference between PRN and fixed group (SMD = 0.17, 95% CI − 0.14–0.48; *p* = 0.28). All three studies individually also reported no significant difference in mean change of CMT. There was no significant heterogeneity between the studies (I^2^ = 26%; *p* = 0.26).

## Discussion

### Summary of main results

The aim of the present meta-analysis is to evaluate the effect of pro re nata and fixed regimen of aflibercept injection for neovascular AMD on visual outcome at 12 months of follow-up. We included four studies in our systematic review and meta-analysis. The result of our meta-analysis shows that fixed aflibercept injection regimen is superior to PRN administration in improving BCVA in neovascular AMD patients. However, the study included in our meta-analysis had high risk of bias, except for one study [11]. Nevertheless, there were low source of heterogeneity in the meta-analysis, meaning that it is possible to estimate the true effect of the treatment. Therefore, we grade the certainty of evidence according to the GRADE certainty rating as moderate certainty.

There was no significant difference in CMT between PRN and fixed group both in individual studies and in pooled analysis. It is established that thicker CMT correlates with worse visual acuity in neovascular AMD patients. This relationship is true both before and after anti-VEGF treatment [15]. Aflibercept’s effectivity to improve CMT, and subsequently BCVA in neovascular AMD patients has been shown to be comparable with ranibizumab [16]. Furthermore, aflibercept injection has also proven to be effective in improving CMT in patients resistant to other anti-VEGF treatment, including ranibizumab and bevacizumab [17]. However, CMT has been shown to be a poor prognostic indicator for long-term visual acuity, with BCVA still considered as the gold standard for treatment evaluation. There are cases where patients had thick CMT but better visual acuity and vice versa [18]. Therefore, although there was no statistically significant difference in CMT change between PRN and fixed group, fixed regimen can still be considered as superior as shown by better BCVA after treatment compared to PRN group.

### Agreements and disagreements with other studies

Fixed regimen of bimonthly aflibercept has been the standard of care for patients with neovascular AMD. The VEGF Trap-EYE Investigation of Efficacy and Safety in Wet AMD (VIEW) studies investigating the efficacy of aflibercept in four treatment group (0.5 mg per 4 weeks, 2 mg per 8 weeks, 2 mg per 8 weeks after 3 loading doses, and 0.5 mg ranibizumab per 4 weeks) shows that mean change in BCVA between the groups were equal, meaning that 2 mg of aflibercept bimonthly is non-inferior compared to ranibizumab. Additionally, eyes treated with aflibercept also achieved higher rate of dryness compared to ranibizumab [19].

Pro re nata regimen for aflibercept has been shown to be effective in treating neovascular AMD. A study by Muftuoglu et al. [20] shows that in patients treated with Aflibercept 2 mg/0.05 cc as needed (indicated by recurrence of intraretinal fluid (IRF) and/or subretinal fluid (SRF), new onset macular hemorrhage, or evidence of any vision loss) to have better anatomic endpoints following 3 consecutive aflibercept injection. Another research by Takayama et al. [21] shows that both single dose pro re nata aflibercept injection and three dose injection significantly improves BCVA, although there was no significant difference in BCVA between single dose and three dose injection.

The result of our study is in accordance to the COPERNICUS study showing that monthly injection of 2 mg aflibercept (2q4) in addition to PRN aflibercept is superior compared to only PRN aflibercept alone, with BCVA in 2q4 + PRN significantly higher compared to sham + PRN (+ 17.3 vs − 4.0, *p* < 0.001 in 24 weeks, + 16.2 vs. + 3.8 letters; *p* < 0.001 in 52 weeks) [22].

### Overall completeness and quality of evidence

We included four studies in our meta-analysis, with one study [11] being a retrospective cohort study. Weber et al. [12] and Veritti et al. [14] included sample size calculation, which shows that their trials were sufficiently powered to detect difference in BCVA between bimonthly and PRN injection.

Unfortunately, all three trials had high risk of bias. The source of the bias is mostly from blinding due to the nature of the intervention being injection. Furthermore, as the outcome measured is best corrected visual acuity, blinding bias, both for subjects and outcome assessor has high probability to influence the outcome. Therefore, future trials that have more rigorous blinding method should be conducted to minimize this bias.

It is important to note that a newer regimen of anti-VEGF dosing for neovascular AMD, that is the Treat and Extend (T&E), has gained its popularity. This regimen combined fixed and PRN dosing, in which patients will receive fixed monthly injection until they reach remission and then proceed to an increase of injection intervals if they continue remission or decrease of injection intervals if there is a relapse. Additional file 1: Table S2 described the outcomes of RCTs on T&E regimen of aflibercept for neovascular AMD [23–26]. Overall, higher mean change of BCVA from baseline was seen in this regimen (+ 7.8 to + 15.9 ETDRS letters) compared to our findings. The total number of injections received in a year was at a range of 6.96 to 8.64. A meta-analysis by Rosenberg et al. [27] showed that at 1 year of follow-up, improvement of VA was similar between T&E and fixed regimen (*p* = *0.95*) and significantly higher in T&E when compared to PRN regimen (*p* < 0.0001). In addition, meta-analysis by Matonti et al. [28] showed that mean change of BCVA and central retinal thickness was similar between fixed and T&E regimen, which was superior to PRN regimen. The total number of injections needed was significantly lower in T&E regimen compared to fixed regimen (8.2 vs. 10.6; p < 0.0001). Nonetheless, subgroup analysis according to the type of anti-VEGF administered was not conducted in both studies. Moreover, there were only two included RCTs on aflibercept that compares T&E with fixed regimen, which were the one written by Haga et al. [23] and Mitchell et al. [24], and none comparing T&E and PRN regimen. Hence, valuable conclusion may not be able to be drawn from these studies.

### Biological plausibility

Aflibercept works as a longer, more stable VEGF-inhibitor. Trials for aflibercept shows that clinical action of aflibercept is approximately 2.5 months, compared to ranibizumab, which has clinical action of approximately 30 days [29]. This allows for bimonthly injection of aflibercept, compared to monthly injection of ranibizumab, which both increases patient compliance and reduce costs [30].

The possible reason of the inferiority of PRN injection compared to bimonthly dosing is due to choroidal neovascularization (CNV) already ongoing even before there is change in BCVA. This means that in the PRN group, it is possible that occult CNV to occur more compared to the bimonthly group, resulting in worse long-term visual acuity. This result is supported by the SUSTAIN study, which shows that retreatment after BCVA loss of > 5 letters, the average gain after retreatment was only 2.6 letters [31]. Additionally, in the post-hoc study by Verriti et al. [14], it is shown that ophthalmologists missed fluid presence in approximately one fourth of fluid-positive scan. This means that it is possible that anatomical indication for PRN aflibercept injection is administered later than expected, which ultimately impact the long-term visual acuity in these patients.

### Applicability of evidence

PRN dosing of aflibercept is still considered as an alternative dosing regimen due to the high cost of bimonthly aflibercept injection. The cost for bimonthly aflibercept injection over a lifetime is approximately $33,745 [31]. Unfortunately, there are no data on the cost-effectiveness of PRN aflibercept injection, therefore it is not known whether PRN aflibercept injection is significantly less cost-intensive compared to bimonthly injection.

### Limitation and potential bias

The present systematic review and meta-analysis is not without limitations. Firstly, we did not find any grey literature on the topic, meaning that publication bias is still possible in our meta-analysis. Secondly, only one study was of low bias, while other studies are high bias, and the biases are likely to influence the result of the studies. Therefore, trials with more rigorous methodology are warranted to eliminate these biases.

### Conclusion

The present meta-analysis shows that bimonthly injection of aflibercept for neovascular AMD is superior compared to PRN injection, shown by better improvement in BCVA at 12 months follow-up. However, high risk of bias downgrade the certainty of evidence.

## Supplementary Information

**Additional file 1: Table S1.** Search strategy for databases.** Table S2. **Summary of RCT outcome in T&E aflibercept regimen for neovascular AMD. **Figure S1.** Sensitivity analysis without retrospective study [11]. **Figure S2.** Sensitivity analysis without studies with high-risk of bias [12, 14].

## Data Availability

Not applicable.
