# Supplementary material for: Pro re nata versus fixed aflibercept regimen for neovascular age-related macular degeneration: a systematic review and meta-analysis
Source: Int J Retina Vitreous. 2022 Sep 22;8:67. doi: 10.1186/s40942-022-00416-x (PMC9503225; doi:10.1186/s40942-022-00416-x)

**Additional files**

**Table S1.** Search strategy for databases

| Database | Search Terms | Hits |
| --- | --- | --- |
| PUBMED | (((aflibercept[Title/Abstract]) AND (pro re nata[Title/Abstract])) AND (macular degeneration[Title/Abstract])) OR (((aflibercept[Title/Abstract]) AND (as needed[Title/Abstract])) AND (macular degeneration[Title/Abstract])) | 117 |
| SCOPUS | ( ( TITLE-ABS-KEY ( pro AND re AND nata ) OR TITLE-ABS-KEY ( prn ) OR TITLE-ABS-KEY ( as AND needed ) ) ) AND ( TITLE-ABS-KEY ( aflibercept ) ) AND ( TITLE-ABS-KEY ( macular AND degeneration ) ) AND ( ( TITLE-ABS-KEY ( retrospective ) OR TITLE-ABS-KEY ( trial ) ) ) AND ( LIMIT-TO ( DOCTYPE , "ar" ) ) | 133 |
| Medline | 1 (pro re nata or prn or as needed).ab.  2 (retrospective or trial).ab.  3 aflibercept.mp.  4 Macular Degeneration/  5 1 and 2 and 3 and 4 | 19 |
| EMBASE | 1 (pro re nata or prn or as needed).ab.  2 (retrospective or trial).ab.  3 aflibercept.mp.  4 Macular Degeneration/  5 1 and 2 and 3 and 4 | 62 |
| EBSCOHost | S1 AB aflibercept AND AB macular degeneration  S2 AB trial OR AB retrospective  (AB trial OR AB retrospective) AND (S1 AND S2) | 74 |
| Web of Science | AB=(pro re nata OR prn OR as needed) AND AB=(aflibercept AND macular degeneration) AND AB=(trial) Refined by: LANGUAGES: ( ENGLISH ) | 56 |
| Clinicaltrial.gov | pro re nata aflibercept, Macular Degeneration | 29 |

**Table S2.** Summary of RCT outcome in T&E aflibercept regimen for neovascular AMD

| **Author (year)** | **Country** | **Study**  **design** | **Age**  **(years)*** | **Sample (N)** | **BCVA at baseline (ETDRS)*** | **T&E regimen** | **Mean change BCVA at 12-months (ETDRS)** | **Total injections*** | **Adverse events (%)** |
| --- | --- | --- | --- | --- | --- | --- | --- | --- | --- |
| Ohji  (2020) | Japan | RCT | 73.0±7.9 | 123 | 54.8±13.1 | 2 weeks | +9 | 7.2±0.9 | 0.8 |
|  |  |  | 75.0±8.1 | 123 | 55.3±12.0 | 4 weeks | +8.4 | 6.9±1.0 | 1.6 |
| Mitchell  (2021) | Multiple countries | RCT | 75.5±9.0 | 106 | 60.2±12.1 | Early-start | +7.8 | 7.1±0.8 | 4.4 |
|  |  |  | 76.6±8.7 | 104 | 61.3±10.8 | Late-start | +10.2 | 8.0±0.2 | 2.9 |
| Taipale  (2019) | Finland | RCT | 76.3±8.5 | 26 | 51.5±15.5 | Moderate extension | +10.3 | 8.64±1.58 | 0 |
|  |  |  | 76.6±6.9 | 26 | 49.5±14.5 | Rapid extension | +11.4 | 6.96±0.79 | 0 |
| Haga  (2017) | Japan | RCT | 75.5±6.7 | 21 | 56.9 | 2 weeks | +15.9 | 7.5±1.2 | n/a |

*value in mean±SD

BCVA=Best Corrected Visual Acuity, ETDRS=Early Treatment Diabetic Retinopathy Study letters

**Figure S1.** Sensitivity analysis without retrospective study (Keppi et al. 2017)


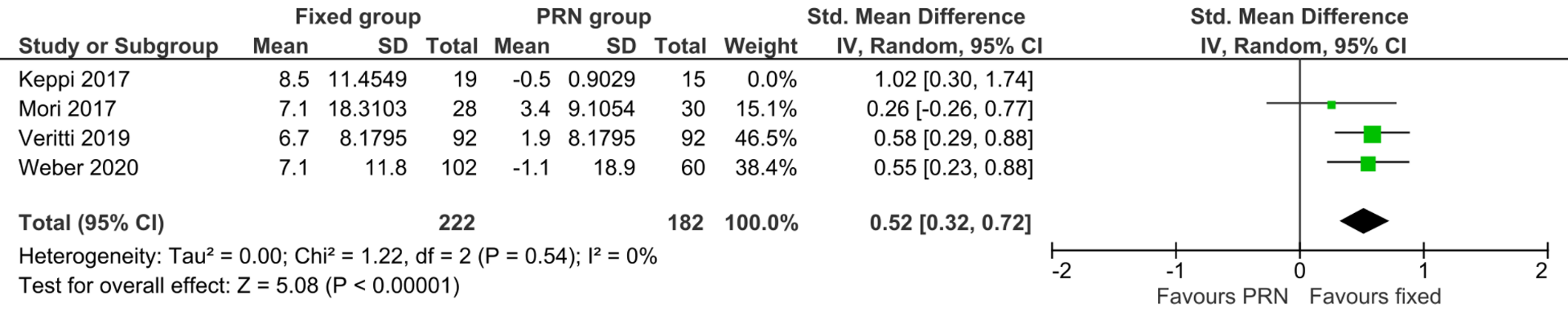


**Figure S2.** Sensitivity analysis without studies with high-risk of bias (Veritti et al. 2019; Weber et al. 2020)


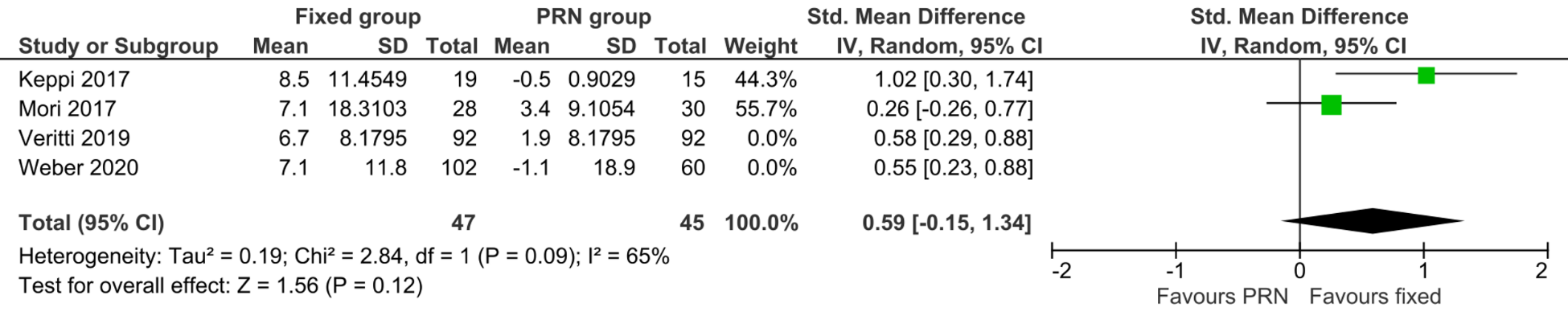

Supplement: Supplementary file 1 — Additional file 1: Table S1. Search strategy for databases. Table S2. Summary of RCT outcome in T&E aflibercept regimen for neovascular AMD. Figure S1. Sensitivity analysis without retrospective study [11]. Figure S2. Sensitivity analysis without studies with high-risk of bias [12, 14]. [file 40942_2022_416_MOESM1_ESM.docx]
